# A novel series of phenolic temozolomide (TMZ) esters with 4 to 5-fold increased potency, compared to TMZ, against glioma cells irrespective of MGMT expression†

**DOI:** 10.1039/d0ra02686g

**Published:** 2020-05-06

**Authors:** Leroy Shervington, Oliver Ingham, Amal Shervington

**Affiliations:** Faculty of Pharmacy and Biomedical Science, The University of Central Lancashire Preston PR1 2HE UK LAShervington@uclan.ac.uk

## Abstract

The standard of care treatment for patients diagnosed with glioblastoma multiforme (GBM) is temozolomide (TMZ). Tumour resistance to TMZ results in significantly limited clinical effectiveness. There is therefore an inherent need for alternatives to TMZ capable of overcoming resistance associated with MGMT and MMR. In the present study, a series of ester and amide analogues of TMZ, modified at position 8 on the imidazole ring, were prepared and investigated for antiproliferative properties. It was found that phenolic ester analogues of TMZ displayed increased potency, of up to 5-fold, against specified glioblastoma cell lines. The encouraging results displayed by the phenolic TMZ esters prompted further investigations against patient-derived primary glioblastoma cultures. The primary cultures, BTNW914 and BTNW374, were MGMT positive and MGMT negative, respectively. Lead phenolic TMZ esters were found to decrease viability in primary cells at clinically relevant concentrations, irrespective of MGMT expression. Furthermore, TMZ was found to be ineffective against the same primary cells at clinically relevant concentrations. The novel phenyl ester analogues of TMZ, described in this study, could have potential chemotherapeutic properties for the treatment of GBM, overcoming the resistance associated with the expression of MGMT.

## Abstract 2

The standard of care treatment for patients diagnosed with glioblastoma multiforme (GBM) is temozolomide (TMZ).

## Graphical abstract

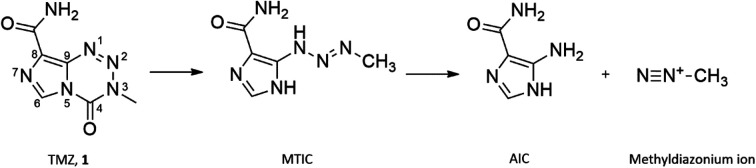

## Introduction

Glioblastoma multiforme (GBM), a grade IV tumour of astrocytic lineage, is the most aggressive glioma in adults accounting for 54% of all diagnosed cases.^1,2^ The most notable advancement in the treatment of GBM in the past 20 years has been the inclusion of the alkylating prodrug, temozolomide (TMZ; 3-methyl-4-oxo-imidazo[5,1-*d*][1,2,3,5]tetrazine-8-carboxamide) in the treatment regimen, resulting in a median survival of 14–15 months.^3,4^

**Fig. 1:**
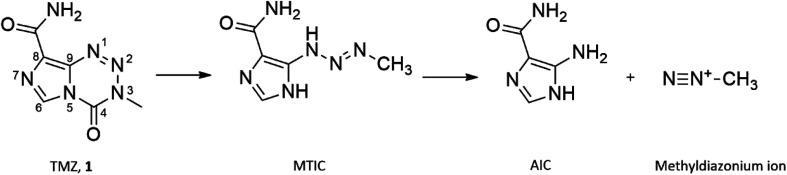
Hydrolytic prodrug activation of TMZ.

Prodrug activation of TMZ occurs through hydrolytic degradation at physiological pH forming the cytotoxic methyldiazonium ion (Fig. 1). The lipophilic nature of the imidazotetrazine core of TMZ allows a more efficient penetration of the blood brain barrier (BBB) compared with other alkylating agents.^5^ TMZ elicits its mechanism of action through the methylation of guanine, forming *O*^6^-methyl guanine (*O*^6^-MeG), causing subsequent mismatch with thymine during replication.^6^ Futile cycles of insertion and deletion of thymine are initiated by mismatch repair (MMR) proteins, which persists until the replication fork collapses, inducing fatal double stranded breaks.^7^ However, 60% of all newly treated GBM patients receive no clinical benefit from treatment with TMZ due to resistance and the development of clinically significant toxicity, attributed to the high dose regimens resulting in further treatment being unsafe.^8,9^ GBM resistance to TMZ treatment is predominantly a result of the expression of the suicide *O*^6^-MeG repair enzyme, *O*^6^-methyl-guanine DNA methyltransferase (MGMT) and the presence of dysfunctional MMR proteins.^10,11^

The aim of the present study was to synthesise novel analogues of TMZ, possessing greater potency than the parent drug. Earlier publications reported on the modifications of imidazotetrazines at position 3 of the molecule, a position that is known to be directly involved in the mechanism of action. However, modifications at this position result in compounds that are either inactive or too toxic for clinical use.^12^ An example of such a compound was the cross-linking 2-chloroethyl analogue, mitozolomide.^12–14^ Interestingly, the amide group present at position 8 of the imidazole ring is not essential for cytotoxicity of TMZ and remains as an artefact from its initial synthesis.^15^ Modifications at position 8 have given rise to analogues with a wide range of activity, however, there is no evidence that supports a structural activity relationship.^15–19^ It is hypothesised that modifications at position 8 could play a role in: modifying the transport properties of the drug; disturbing the interactions between the inactive prodrug and the DNA; or exerting kinetic control of the hydrolytic degradation of the prodrug.^15,20^

In the present study, a series of position 8 modified ester and amide analogues of TMZ were synthesised, with the aim of increasing the activity. The modified analogues could provide the foundations for potential alternatives to TMZ that possess superior chemotherapeutic activity for the treatment of GBM.

## Results and discussion

### Chemistry

**Scheme 1:**
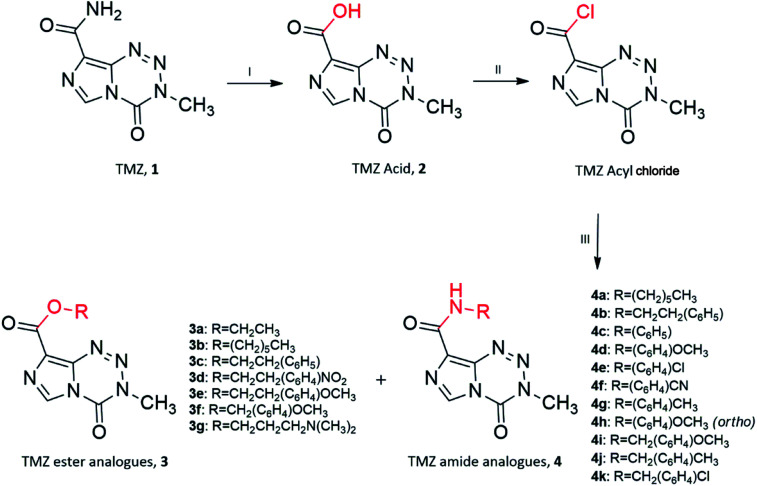
Synthesis of TMZ analogues. (I) NaNO_2_, H_2_SO_4_, <15 °C; (II) SOCl_2_, DMF, reflux; (III) R–OH/R–NH_2_, THF, r.t. Ester and amide analogues are referred to as 3 and 4, respectively.

Ester and amide TMZ analogues were synthesised using two schemes. The general route of synthesis outlined in Scheme 1 was originally described by Horspool *et al.*, (1990).^21^ Ester analogues (3a–3g) and amide analogues (4a–4k) of TMZ were synthesised through hydrolysis of the TMZ carbomoyl group using nitrous acid to form TMZ acid 2 (3-methyl-4-oxo-imidazo[5,1-*d*][1,2,3,5]tetrazine-8-carboxylic acid). Conversion of TMZ acid to TMZ acyl chloride (3-methyl-4-oxo-imidazo[5,1-*d*][1,2,3,5]tetrazine-8-carbonyl chloride) was readily achieved through reflux with thionyl chloride, catalysed by Vilismeier iminium salt formation through the addition of dimethylformamide. Preferential nucleophilic attack of the imidazo carbonyl group on TMZ acyl chloride by the various alcohols and amines, yielded the target analogues.

**Scheme 2:**
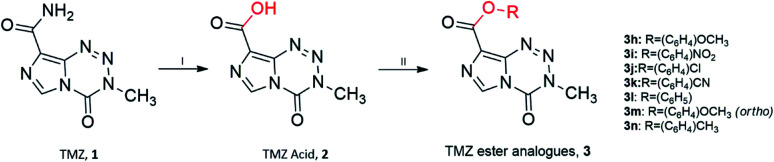
Synthesis of TMZ analogues. (I) NaNO_2_, H_2_SO_4_, <15 °C; (II) EDC·HCl, DMAP, THF : DCM (1 : 1), R–OH/R–NH_2_, r.t. Ester analogues are referred to as 3.

Unfortunately, reactions between TMZ acyl chloride and phenyl alcohols resulted in unfavourable yields, mainly attributed to steric effects. Therefore a combination of the coupling reagent, EDC·HCl (1-(3-dimethylaminopropyl)-3-ethylcarbodiimide hydrochloride) and catalyst, DMAP (4-dimethylaminopyridine) were used to help overcome these effects (Scheme 2).^22^ The water-soluble urea by-product was subsequently removed *via* a simple aqueous work up, yielding ester analogues (3h–3n).

### Cytotoxic evaluation

Apoptosis of glioma cells induced by TMZ, occurs at least 120 hours after treatment.^7^ Consequently, cells were incubated for 144 hours with specified TMZ analogues in the *in vitro* investigations in order to obtain more reliable IC_50_ values.

In order to assess the cytotoxic potential of the synthesised TMZ analogues, initial screening was conducted against the grade IV glioblastoma cell line, U87-MG (Table 1). For TMZ and TMZ acid the average IC_50_ was 696 ± 79 and 662 ± 42 μM, respectively. The equi-cytotoxicity observed between TMZ and TMZ acid against U87-MG cells was consistent with earlier published literature using lymphoma cells (TLX9)^23^ and glioma cells (TJ899; TJ905 and SHG-44).^19^ TMZ analogues 3a, 3c, 3g, 3m and 4b, similar to TMZ acid, exhibited cytotoxicity comparable to or less than TMZ. Analogue 3g, an analogue originally described by Liu *et al.*, (2010), was reported to exhibit promising cytotoxic potential, however, testing did not include glioblastoma cell lines, thus providing the rationale of its inclusion in the present study.^18^ This analogue was found to be less active than TMZ and was therefore removed from the investigation.

**Table 1:** IC_50_ concentrations (μM) of TMZ analogues against U87-MG cells^a^

Analogue	IC_50_^b^ (μM)	Analogue	IC_50_^b^ (μM)
TMZ	696 ± 79	3l	331 ± 47
TMZ acid	662 ± 42	3m	576 ± 91
3a	700 ± 109	3n	376 ± 62
3b	409 ± 45	4a	>200
3c	615 ± 96	4b	548 ± 48
3d	—	4c	>200
3e	—	4d	—
3f	—	4e	—
3g	856 ± 20	4f	—
3h	134 ± 7	4g	—
3i	142 ± 3	4h	—
3j	96 ± 3	4i	—
3k	138 ± 7	4j	139 ± 28
		4k	—

a All the data were the average values of three independent assays, IC_50_ ± SD (*n* = 3).

b Compound concentration that reduces cell viability by 50% compared to untreated cells.

Although attempts to assess the IC_50_ of the analogues against U87-MG were made, a significant proportion of the amide analogues and three of the ester analogues, (3d, 3e, 3f, 4a, 4c, 4d, 4e, 4f, 4g, 4h, 4i, 4k) were found to be insoluble in cell culture medium and were therefore removed from the studies. The maximum concentration that could be achieved in medium for amide analogues 4a and 4c was approximately 200 μM, at which IC_50_ values could not be generated and were also eliminated from further studies. Interestingly, ester analogues (3b, 3h, 3i, 3j, 3k, 3l, 3n) exhibited 2 to 5-fold greater activity against the U87-MG cell lines compared to TMZ and were subsequently taken forward to test against additional cell lines. Interestingly, the phenyl containing ester analogues 3h, 3i, 3j, 3k, 3l and 3n were found to be the most effective when tested against the U87-MG cell line, compared with TMZ. Due to TMZ hexyl ester 3b, initially described by Suppasansatorn as a compound for topical treatment of melanoma,^19^ was reported to exhibit promising cytotoxic potency against melanoma cells, the analogue was tested against glioblastoma cell lines. The ester 3b exhibited significantly increased cytotoxic potency against U87-MG cells compared to TMZ (IC_50_ = 409 μM).

The ester analogues, (3d, 3e, 3f, 4a, 4c, 4d, 4e, 4f, 4g, 4h, 4i, 4k) were found to be insoluble in cell culture medium and were therefore removed from the studies.

Due to the encouraging cytotoxicity results of analogues 3b, 3h, 3i, 3j, 3k, 3l, and 3n against the U87-MG cell line, further testing was carried out against 1321-N1, GOS-3 and the normal SVGp12 cell line in order to gain a more in-depth understanding of their cytotoxic potency against various grades of glioma, as well as non-malignant glial cells (Table 2). The most promising compounds found through the cytotoxic evaluation were the ester analogues consisting of *para*-substituted aromatic moieties, containing methoxy, nitro, chloro and nitrile groups (3h, 3i, 3j and 3k), which exhibited activity 4 to 5-fold greater than TMZ across each of the cell lines used. The introduction of an *ortho*-substituted methoxy moiety (analogue 3m) in place of the corresponding *para*-substituted methoxy moiety (analogue 3h) resulted in reduced activity. Additionally, the unsubstituted phenyl analogue 3l showed less cytotoxic potential than its substituted counter-parts (3h, 3i, 3j and 3k). Interestingly, although analogue 3n, containing a methyl moiety in the *para*-position, displayed greater activity than analogue 3l, it was significantly less active than analogues containing more polarising aromatic substituents.

**Table 2:** IC_50_ concentrations (μM) of lead TMZ analogues against U87-MG, 1321-N1, GOS-3 and the normal SVGp12 cell line^a^

Analogue	IC_50_^b^ (μM)			
	U87-MG	1321-N1	GOS-3	SVGp12
TMZ	621 ± 66	783 ± 40	696 ± 16	239 ± 6
TMZ acid	662 ± 42	596 ± 26	504 ± 30	316 ± 2
3b	409 ± 45	269 ± 20	269 ± 6	182 ± 11
3h	134 ± 7	183 ± 1	183 ± 5	44 ± 2
3i	141 ± 3	104 ± 4	91 ± 1	39 ± 2
3j	96 ± 3	118 ± 11	54 ± 4	33 ± 1
3k	137 ± 7	103 ± 7	151 ± 4	36 ± 3
3l	330 ± 47	511 ± 10	691 ± 35	238 ± 6
3n	376 ± 62	238 ± 8	314 ± 38	215 ± 26
4k	138 ± 28	>200	>200	79 ± 6

a All the data were the average values of three independent assays, IC_50_ ± SD (*n* = 3).

b Compound concentration that reduces cell viability by 50% compared to untreated cells.

The mean plasma concentration of TMZ after oral ingestion of a 200 mg tablet is between 70–80 μM.^24,25^ Brain-tumour concentrations of TMZ are estimated to be approximately 20% of the plasma concentration, equivalent to a concentration of 15 μM.^25,26^ However, because TMZ is a prodrug and is not biologically active until it degrades to AIC and the cytotoxic methyldiazonium ion, these concentrations may not accurately reflect the level of the active drug within the region of the tumour. These levels are likely to be higher than what is observed in these studies since a mixture of TMZ, MTIC, and methyldiazonium ions will be present in the vicinity of the tumour, all of which have cytotoxic properties or cytotoxic potential.^25^ An evaluation of the literature infers that a clinically relevant concentration of TMZ ranges from approximately 15 to 75 μM.

At the clinically relevant concentrations of 15 and 75 μM, TMZ was found to reduce the viability of MGMT devoid cells by approximately 20%. The relatively modest reduction in viability supports the findings that patients treated with TMZ gain a modest therapeutic benefit.^8^ Our results clearly show that TMZ is only effective against MGMT devoid GBM cells at concentrations that are not clinically relevant following a 200 mg m^−2^ oral dose. Interestingly, analogues containing the methoxy, nitro, choro and nitrile phenyl esters (3h, 3i, 3j and 3k) generate IC_50_'s at concentrations clinically relevant in the tumour (<15 μM), thus, suggesting these analogues could have a potential to generate a significant clinical effect in patients suffering from GBM devoid of MGMT, compared to TMZ. The phenyl ester analogues 3l and 3n were also found to be significantly more effective than TMZ, inducing a significant reduction in viability greater than 50% at 75 μM against MGMT devoid GBM cells. However, these two analogues were found to be less potent than the analogues containing highly polarising groups (3h, 3i, 3j and 3k).

**Fig. 2:**
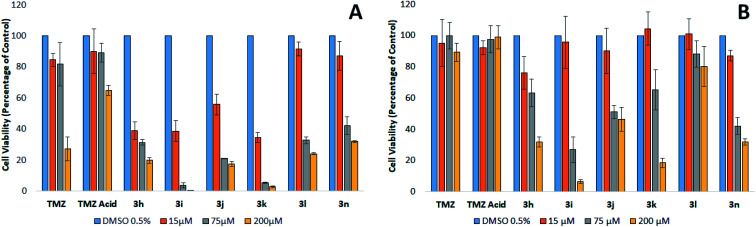
(A) Cell viability of BTNW914 (MGMT−) after treatment by TMZ and phenyl TMZ ester analogues at various clinically relevant concentrations and incubation for 6 days. (B) Cell Viability of BTNW374 (MGMT+) after treatment by TMZ and phenyl TMZ ester analogues at various clinically relevant concentrations. Values are reported as the percentage of viable cells ± SD, *n* = 3.

Fig. 2 shows the cytotoxic potency of the most promising analogues, (3h, 3i, 3j, 3k and 3n) against BTNW374, an MGMT positive expressed patient derived primary cultures. As expected, the repair of the cytotoxic *O*^6^-methylation by MGMT appeared to cause TMZ resistance. The MGMT expressing cells also demonstrated similar resistance to TMZ acid and phenyl ester 3l, suggesting that these analogues possibly generate cytotoxicity *via* a similar mechanism to that of TMZ. Interestingly, the 4-methoxy, 4-nitro, 4-chloro, 4-nitrile and *p*-cresol phenyl esters (3h, 3i, 3j, 3k and 3n respectively), generated cytotoxicity at 75 μM, independent of the MGMT status. Since the imidazotetrazine ring remains unmodified in these analogues, it is plausible to assume that methylation still contributes to cytotoxicity in TMZ sensitive cells. Furthermore, it has recently been established that modifications at position 8 of the imidazole ring can influence the rate of prodrug activation, therefore, it would be reasonable to suggest that modifying the substituent on the phenyl ester moiety could possibly effect the rate at which these prodrugs produce methyl diazonium ions.^10,15^ However, as these analogues retain activity against TMZ resistant cells that express, it is plausible to conclude that an alternate mechanism may be involved, distinct from methylation. In the experiments using patient derived primary cultures expressing high levels of MGMT, analogues 3h, 3i, 3j, 3k and 3n were found to be significantly more effective compared to TMZ. This evidence suggests that these analogues should be further investigated as potential chemotherapeutic agents for the treatment of GBM, irrespective of the MGMT status of the tumour.

### The stability of lead phenolic TMZ analogues after exposure to porcine liver esterase (PLE)

The hydrolysis was monitored by the decrease in the peak area of the corresponding TMZ esters, however, due to sensitivity of the imidazotetrazine ring to pH, the experiments were carried out at pH 6 to avoid the formation of imidazoles. The validation of the analytical method also involved investigating the stability of the imidazotetrazine ring whilst ensuring PLE retained a significant level of activity. These findings were in keeping with the work published by Junge and Heymann (1979), who found that PLE has a broad pH optima (pH 6–8).^27^

Results depicted in Table 3 indicate that 50% of each of the TMZ esters were hydrolysed by PLE in the first 14 minutes of exposure. In fact, 50% of TMZ esters containing *para*-nitro, chloro, phenyl and tolyl groups (3i, 3j, 3l and 3n), were hydrolysed within the first 4.2 minutes of PLE exposure (Table 3). TMZ esters containing *para*-methoxy and nitrile groups (3h and 3k) conferred increased stability, with 50% hydrolysis being achieved in 7.3 and 13.7 minutes, respectively. The maximum intracranial concentration of TMZ is reported to be within a time frame of approximately 2 ± 0.8 hours.^26^

**Table 3:** The time taken for PLE to hydrolyse 50% of phenolic TMZ esters

Analogue	3h	3i	3j	3k	3l	3n
Time taken to hydrolyse 50% of the ester (minutes)	7.3	4.2	4.1	13.7	3.4	3.2

As a result, it is unlikely that the lead phenolic TMZ esters would remain intact on reaching the site of a brain tumour, due to the rapid rate of hydrolysis observed. Thus, it is probable that TMZ esters would be hydrolysed to TMZ acid and the corresponding alcohol. Therefore, the increased activity observed *in vitro* could potentially be lost. As a result, these findings pose a question regarding the effectiveness of all 6 esters in an *in vivo* setting. However, despite the obvious challenges the ester bond poses, the exceptional *in vitro* activity displayed, irrespective of MGMT expression by these analogues (3h, 3i, 3j, 3k, 3l and 3n) should be further investigated.

## Experimental

### General

All solvents and reagents were used without any further purification. Reactions were monitored using silica gel coated TLC plates with fluorescent indicator (254 nm). Flash column chromatography, used for purification, was carried out using silica gel purchased from Sigma Aldrich. Specifications of the silica gel were; 60 Å pore size, 230–400 mesh particle size and 40–63 μm particle size. ^1^H and ^13^C NMR were recorded on either a Bruker Fourier 300 (300 MHz) or a Bruker Advanced III 400 (400 MHz) and interpreted using Mestrenova software. All chemical shifts are relative to residual solvent peaks (chloroform-*d* CDCl_3_, *δ*_H_-7.26; *δ*_C_-77.16); (DMSO-*d*_6_, *δ*_H_-2.50; *δ*_C_-39.52). *J* Values are calculated in Hz. IR spectra were recorded on Nicolet iS10 Spectrometer and interpreted using OMNICs software. Melting points were recorded on a Stuart SMP10 melting point apparatus and are uncorrected. HRMS to 4 dp was obtained by Pharmidex by means of UHPLC-ToF-MS using electrospray ionisation on an Agilent 1290 UHPLC and Agilent 6550 QToF-MS.

### Synthesis of 3-methyl-4-oxo-3,4-dihydroimidazo[5,1-*d*][1,2,3,5]tetrazine-8-carboxylic acid (2)

TMZ 1 (2.00 g, 10.3 mmol) was dissolved in concentrated sulphuric acid (16 mL). Sodium nitrite (2.65 g, 38.4 mmol) in water (10.4 mL) was added dropwise to the solution, keeping the temperature of the reaction mixture below 15 °C. The resulting mixture was stirred for 16 hours. Ice water (40 mL) was added to the mixture, precipitating out the desired product. 3-Methyl-4-oxo-imidazo[5,1-*d*][1,2,3,5]tetrazine-8-carboxylic acid (2, TMZ acid) was collected *via* filtration before being dried over phosphorus pentoxide in a desiccator for 48 hours. Producing a white solid, 1.36 g (68% yield). Mp 181 °C (decomposition); IR (cm^−1^) 3499 (O–H stretch), 3080 (C–H stretch), 1764 (C=O stretch, COOH), 1686 (C=O stretch, CONH), ^1^H NMR (300 MHz, DMSO-*d*_6_), *δ*_H_: 13.40 (bs, 1H, OH), 8.82 (s, 1H, CH), 3.87 (s, 3H, CH_3_). ^13^C NMR (75 MHz, DMSO-*d*_6_), *δ*_C_: 162.28, 139.52, 136.91, 129.52, 128.17, 36.76. HRMS calcd for C_6_H_5_N_5_O_3_ ([M + Na]^+^): 218.0284; found *m*/*z*: 218.0293.

### Synthesis of analogues 3a–3g and 4a–4k

A mixture of TMZ acid (2, 200 mg, 1.03 mmol), thionyl chloride (5 mL, 69 mmol) and DMF (1 drop) was heated under reflux for 3 hours. The resulting mixture was evaporated under reduced pressure. Toluene (3 × 10 mL) was added and evaporated to dryness to yield, 3-methyl-4-oxo-imidazo[5,1-*d*][1,2,3,5] tetrazine-8-carbonyl chloride (TMZ acyl chloride), as a brown/orange solid 218 mg, (99% yield). TMZ acyl chloride (218 mg, 1.02 mmol) was dissolved in tetrahydrofuran (10 mL). A solution of tetrahydrofuran (2 mL) and the appropriate alcohol or amine (1.05 mmol) was added dropwise and stirred for 3 hours. The crude reaction mixture was embedded onto silica, and subjected to column chromatography using a solvent system of an appropriate ratio of petroleum ether (40–60 °C) to ethyl acetate, to afford the desired title compounds.

#### Ethyl 3-methyl-4-oxo-3,4-dihydroimidazo[5,1-*d*][1,2,3,5]tetrazine-8-carboxylate (3a)

Light orange solid 145.0 mg (79% yield). Mp. 130 °C; IR (cm^−1^) 1716 (C=O stretch, COO), 1623 (C=O stretch, CONH). ^1^H NMR (300 MHz, DMSO-*d*_6_), *δ*_H_: 8.85 (s, 1H, CH), 4.38 (q, *J* = 7.1 Hz, 2H, CH_2_), 3.88 (s, 3H, CH_3_), 1.33 (t, *J* = 7.1 Hz, 3H, CH_3_). ^13^C NMR (75 MHz, DMSO-d_6_), *δ*_C_: 160.9, 139.4, 137.1, 129.8, 127.1, 61.3, 36.9, 14.7. HRMS calcd for C_8_H_9_N_5_O_3_ ([M + Na]^+^): 246.0598; found *m*/*z*: 246.0629.

#### Hexyl 3-methyl-4-oxo-3,4-dihydroimidazo[5,1-*d*][1,2,3,5]tetrazine-8-carboxylate (3b)

Yellow solid 81.2 mg (29% yield). Mp. 76 °C; IR (cm^−1^) 3114 (C–H stretch), 2956 (C–H stretch), 2921 (C–H stretch), 2854 (C–H stretch), 1755 (C=O stretch, COO), 1722 (C=O stretch, CONH). ^1^H NMR (300 MHz DMSO-*d*_6_), *δ*_H_: 8.85 (s, 1H, CH), 4.34 (t, *J* = 7.1 Hz, CH_2_), 3.88 (s, 3H, CH_3_), 1.71 (quint, *J* = 7.1 Hz, 2H, CH_2_), 1.53–1.16 (m, 8H, (CH_2_)_4_), 0.87 (t, *J* = 7.1 Hz, 3H, CH_3_). ^13^C NMR (75 MHz DMSO-*d*_6_), *δ*_C_: 161.0, 139.4, 137.1, 129.8, 127.1, 65.2, 36.8, 31.3, 28.6, 25.5, 22.5, 14.4. HRMS calcd for C_12_H_17_N_5_O_3_ ([M + Na]^+^): 302.1224; found *m*/*z*: 302.1239.

#### Phenethyl 3-methyl-4-oxo-3,4-dihydroimidazo[5,1-*d*][1,2,3,5]tetrazine-8-carboxylate (3c)

Off-white solid 177.4 mg (44% yield). Mp 124 °C; IR (cm^−1^) 3067 (C–H stretch), 1740 (C=O stretch, COO), 1712 (C=O stretch, CONH). ^1^H NMR (300 MHz DMSO-*d*_6_), *δ*_H_: 8.85 (s, 1H, CH), 7.38–7.19, (m, 5H, ArH), 4.55 (t, *J* = 7.5 Hz, 2H, CH_2_), 3.89 (s, 3H, CH_3_), 3.05 (t, *J* = 7.5 Hz, 2H, CH_2_). ^13^C NMR (75 MHz DMSO-*d*_6_), *δ*_C_: 160.9, 139.4, 138.4, 137.2, 129.8, 129.5, 128.8, 126.9, 126.9, 65.8, 36.9, 34.9. HRMS calcd for C_14_H_13_N_5_O_3_ ([M + Na]^+^): 322.0911; found *m*/*z*: 322.0925.

#### 4-Nitrophenethyl 3-methyl-4-oxo-3,4-dihydroimidazo[5,1-*d*][1,2,3,5]tetrazine-8-carboxylate (3d)

White solid 102.6 mg (27% yield). Mp 187 °C; IR (cm^−1^) 3105 (C–H stretch), 1743 (C=O stretch, COO), 1708 (C=O stretch, CONH). ^1^H NMR (300 MHz, DMSO-*d*_6_), *δ*_H_: 8.86 (s, 1H, CH), 8.15 (d, *J* = 8.3 Hz, 2H, ArH), 7.67 (d, *J* = 8.3 Hz, 2H, ArH), 4.63 (t, *J* = 6.4 Hz, 2H, CH_2_), 3.90 (s, 3H, CH_3_), 3.22 (t, *J* = 6.4 Hz, 2H, CH_2_). ^13^C NMR (75 MHz DMSO-*d*_6_), *δ*_C_: 160.7, 147.0, 146.7, 139.4, 137.2, 130.9, 129.9, 126.7, 123.8, 65.0, 36.9, 34.6. HRMS calcd for C_14_H_12_N_6_O_3_ ([M + Na]^+^): 367.0761; found *m*/*z*: 367.0772.

#### 4-Methoxyphenethyl 3-methyl-4-oxo-3,4-dihydroimidazo[5,1-*d*][1,2,3,5]tetrazine-8-carboxylate (3e)

Off-white solid 79 mg (22% yield). Mp 164 °C; IR (cm^−1^) 3126 (C–H stretch), 1745 (C=O stretch, COO), 1725 (C=O stretch, CONH). ^1^H NMR (300 MHz, DMSO-*d*_6_), *δ*_H_: 8.85 (s, 1H, CH), 7.27 (d, *J* = 8.4 Hz, 2H, ArH), 6.85 (d, *J* = 8.4 Hz, 2H, ArH), 4.48 (t, *J* = 6.8 Hz, 2H, CH_2_), 3.89 (s, 3H, CH_3_), 3.71 (s, 3H, CH_3_), 2.98 (t, *J* = 6.8 Hz, 2H, CH_2_). ^13^C NMR (75 MHz DMSO-*d*_6_), *δ*_C_: 160.9, 158.3, 139.4, 137.2, 130.5, 130.1, 129.8, 127.0, 114.2, 66.1, 55.4, 36.9, 34.0. HRMS calcd for C_15_H_15_N_5_O_4_ ([M + Na]^+^): 352.1016; found *m*/*z*: 352.1029.

#### 4-Methoxybenzyl 3-methyl-4-oxo-3,4-dihydroimidazo[5,1-*d*][1,2,3,5]tetrazine-8-carboxylate (3f)

Yellow solid 111.4 mg (37% yield); mp 170 °C, IR (cm^−1^) 3122 (C–H stretch), 2961 (C–H stretch), 2928 (C–H stretch), 1752 (C=O stretch, COO), 1719 (C=O stretch, CONH). ^1^H NMR (300 MHz, DMSO-*d*_6_), *δ*_H_: 8.85 (s, 1H, CH), 7.44 (d, *J* = 8.4 Hz, 2H, ArH), 6.96 (d, *J* = 8.4 Hz, 2H, ArH), 5.36 (s, 2H, CH_2_), 3.88 (s, 3H, CH_3_), 3.76 (s, 3H, CH_3_). ^13^C NMR (75 MHz DMSO-*d*_6_), *δ*_C_: 160.8, 159.7, 139.4, 137.3, 130.5, 129.9, 128.3, 126.9, 114.3, 66.4, 55.6, 36.9. HRMS calcd for C_14_H_13_N_5_O_4_ ([M + Na]^+^): 338.0860; found *m*/*z*: 338.8075.

#### 3-(Dimethylamino)propyl 3-methyl-4-oxo-3,4-dihydroimidazo[5,1-*d*][1,2,3,5]tetrazine-8-carboxylate (3g)

White solid 132.4 mg (46% yield). Mp 204 °C, IR (cm^−1^) 3074 (C–H), 2595–2483 (C–H), 1747 (C=O stretch, COO), 1719 (C=O stretch, CONH), 1615–692. ^1^H NMR (300 MHz, DMSO-*d*_6_) *δ*_H_: 8.89 (s, 1H, CH), 4.43 (t, *J* = 6.2 Hz, 2H, CH_2_), 3.89 (s, 3H, CH_3_), 3.20(t, *J* = 6.2 Hz, 2H, CH_2_), 2.75 (s, 6H, CH_3_), 2.18 (quint, *J* = 6.2 Hz, 2H, CH_2_). ^13^C NMR (100 MHz, DMSO) *δ*_C_: 160.2, 138.9, 136.8, 129.4, 126.3, 62.1, 53.8, 42.1, 36.4, 23.3. HRMS calcd for C_14_H_13_N_5_O_4_ ([M + H]^+^): 281.1357; found *m*/*z*: 281.1372.

#### *N*-Hexyl-3-methyl-4-oxo-3,4-dihydroimidazo[5,1-*d*][1,2,3,5]tetrazine-8-carboxamide (4a)

Off-white solid 156.5 mg (55% yield). Mp 138 °C; IR (cm^−1^), 3295 (N–H stretch), 3118 (C–H stretch), 2921 (C–H stretch), 2856 (C–H stretch), 1726 (C=O stretch, CONH), 1652 (C=O stretch, CONH). ^1^H NMR (400 MHz, chloroform-*d*), *δ*_H_: 8.40 (s, 1H, CH), 7.35 (t, *J* = 6.5 Hz, 1H, NH), 4.04 (s, 3H CH_3_), 3.51 (q, *J* = 6.5 Hz, 2H, CH_2_), 1.63 (quint, *J* = 6.5 Hz, 2H, CH_2_), 1.46–1.27 (m, 6H, (CH_2_)_3_), 0.89 (t, *J* = 6.5 Hz, 3H, CH_3_). ^13^C NMR (100 MHz chloroform-*d*), *δ*_C_: 159.3, 138.8, 133.7, 132.1, 127.7, 39.4, 36.5, 31.5, 29.5, 22.53, 14.0. HRMS calcd for C_12_H_18_N_6_O_2_ ([M + Na]^+^): 301.1384; found *m*/*z*: 301.1409.

#### 3-Methyl-4-oxo-*N*-phenethyl-3,4-dihydroimidazo[5,1-*d*] [1,2,3,5]tetrazine-8-carboxamide (4b)

Off-white solid 169.2 mg (58% yield). Mp 158 °C; IR (cm^−1^) 3312 (N–H stretch), 3082 (C–H stretch), 1745 (C=O stretch, CONH), 1646 (C=O stretch, CONH). ^1^H NMR (400 MHz, DMSO-*d*_6_), *δ*_H_: 8.84 (s, 1H, CH), 8.54 (t, *J* = 5.9 Hz, 1H, NH), 7.36–7.16 (m, 5H, ArH), 3.87 (s, 3H, CH_3_), 3.54 (q, *J* = 7.4 Hz, 2H, CH_2_), 2.87 (t, *J* = 7.4 Hz, 2H, CH_2_). ^13^C NMR (100 MHz DMSO-*d*_6_), *δ*_C_: 160.0, 139.8, 139.7, 134.9, 130.8, 129.1, 128.9, 128.8, 126.6, 40.6, 36.6, 35.6. HRMS calcd for C_14_H_14_N_6_O_2_ ([M + Na]^+^): 321.1071; found *m*/*z*: 321.1088.

#### 3-Methyl-4-oxo-*N*-phenyl-3,4-dihydroimidazo[5,1-*d*][1,2,3,5]tetrazine-8-carboxamide (4c)

Light orange solid 110.3 mg (40% yield). Mp 189 °C; IR (cm^−1^) 3356 (N–H stretch), 3118 (C–H stretch), 1734 (C=O stretch, CONH), 1684 (C=O stretch, CONH). ^1^H NMR (300 MHz, DMSO-*d*_6_), *δ*_H_: 10.40 (s, 1H, NH), 8.96 (s, 1H, CH), 7.88 (d, *J* = 6.0 Hz, 2H, ArH), 7.36 (t, *J* = 6.0 Hz, 2H, ArH), 7.15 (t, *J* = 6.0 Hz, 1H, ArH), 3.89 (s, 3H, CH_3_). ^13^C NMR (75 MHz, DMSO-*d*_6_), *δ*_C_: 158.8, 139.6, 138.9, 135.6, 130.6, 129.1, 129.0, 124.4, 120.7, 36.7. HRMS calcd for C_12_H_10_N_6_O_2_ ([M + Na]^+^): 293.0757; found *m*/*z*: 293.0772.

#### *N*-(4-Methoxyphenyl)-3-methyl-4-oxo-3,4-dihydroimidazo[5,1-*d*][1,2,3,5]tetrazine-8-carboxamide (4d)

Yellow solid 75.6 mg (25% yield). Mp 192 °C; IR (cm^−1^) 3376 (N–H stretch), 3119 (C–H stretch), 2937 (C–H stretch), 1746 (C=O stretch, CONH), 1684 (C=O stretch, CONH). ^1^H NMR (300 MHz, DMSO-*d*_6_), *δ*_H_: 10.30 (s, 1H, NH), 8.95 (s, 1H, CH), 7.79 (d, *J* = 9.1 Hz, 2H, ArH), 6.94 (d, *J* = 9.1 Hz, 2H, ArH), 3.89 (s, 3H, CH_3_), 3.75 (s, 3H, CH_3_). ^13^C NMR (75 MHz, DMSO-*d*_6_), *δ*_C_: 158.4, 156.1, 139.7, 135.5, 132.0, 130.8, 128.9, 122.3, 114.2, 55.6, 36.7. HRMS calcd for C_13_H_12_N_6_O_3_ ([M + Na]^+^): 323.0863; found *m*/*z*: 323.0877.

#### *N*-(4-Chlorophenyl)-3-methyl-4-oxo-3,4-dihydroimidazo[5,1-*d*][1,2,3,5]tetrazine-8-carboxamide (4e)

Brown solid 140.7 mg (45% yield). Mp 200 °C; IR (cm^−1^) 3359 (N–H stretch), 3117 (C–H stretch), 1737 (C=O stretch, CONH), 1689 (C=O stretch, CONH). ^1^H NMR (400 MHz, DMSO-*d*_6_), *δ*_H_: 10.59 (s, 1H, NH), 8.97 (s, 1H, CH), 7.93 (d, *J* = 8.8 Hz, 2H, ArH), 7.42 (d, *J* = 8.8 Hz, 2H, ArH), 3.90 (s, 3H, CH_3_). ^13^C NMR (100 MHz, DMSO-*d*_6_), *δ*_C_: 158.4, 139.1, 137.4, 135.3, 129.8, 128.5, 127.5, 121.8, 36.2. HRMS calcd for C_12_H_9_N_6_O_2_Cl ([M + Na]^+^): 327.0368; found *m*/*z*: 327.0382.

#### *N*-(4-Cyanophenyl)-3-methyl-4-oxo-3,4-dihydroimidazo[5,1-*d*][1,2,3,5]tetrazine-8-carboxamide (4f)

Off-white solid 216.0 mg (73% yield). Mp 234 °C; IR (cm^−1^) 3339 (N–H stretch), 3117 (C–H stretch), 2222 (C≡N stretch), 1740 (C=O stretch, CONH), 1689 (C=O stretch, CONH). ^1^H NMR (400 MHz, DMSO-*d*_6_), *δ*_H_: 10.75 (s, 1H, NH), 8.93 (s, 1H, CH), 8.10 (d, *J* = 8.8 Hz, 2H, ArH), 7.81 (d, *J* = 8.8 Hz 2H, ArH), 3.90 (s, 3H, CH_3_). ^13^C NMR (100 MHz, DMSO-*d*_6_), *δ*_C_: 158.8, 142.7, 139.0, 135.5, 133.0, 129.5, 128.6, 120.3, 118.9, 105.7, 36.2. HRMS calcd for C_13_H_9_N_7_O_2_ ([M + Na]^+^): 318.0710; found *m*/*z*: 318.0720.

#### 3-Methyl-4-oxo-*N*-(*p*-tolyl)-3,4-dihydroimidazo[5,1-*d*][1,2,3,5]tetrazine-8-carboxamide (4g)

Yellow solid 164.7 mg (58%). Mp 209 °C; IR (cm^−1^) 3301 (N–H stretch), 3114 (C–H stretch), 1735 (C=O stretch, CONH), 1672 (C=O stretch, CONH). ^1^H NMR (400 MHz, DMSO-*d*_6_) *δ*_H_: 10.30 (s, 1H, NH), 8.94 (s, 1H, CH), 7.75 (d, *J* = 8.8 Hz, 2H, ArH), 7.16 (d, *J* = 8.8 Hz, 2H, ArH), 3.89 (s, 3H, CH_3_), 2.28 (s, 3H, CH_3_). ^13^C NMR (100 MHz, DMSO) *δ*_C_: 158.6, 139.6, 136.4, 135.5, 133.4, 130.7, 129.5, 128.9, 120.7, 36.7, 21.0. HRMS calcd for C_13_H_12_N_6_O_2_ ([M + Na]^+^): 307.0914; found *m*/*z*: 307.0929.

#### *N*-(2-Methoxyphenyl)-3-methyl-4-oxo-3,4-dihydroimidazo[5,1-*d*][1,2,3,5]tetrazine-8-carboxamide (4h)

Yellow solid 268.7 mg (87%). Mp 193 °C; IR (cm^−1^) 3370 (N–H stretch), 1741 (C=O stretch, CONH), 1677 (C=O stretch, CONH). ^1^H NMR (400 MHz, DMSO-*d*_6_) *δ*_H_: 9.78 (s, 1H, NH), 8.95 (s, 1H, CH), 8.40 (d, *J* = 8.8 Hz, 1H, ArH), 7.14 (d, *J* = 8.8 Hz, 2H, ArH), 7.01 (m, 1H, ArH), 3.94 (s, 3H, CH_3_), 3.90 (s, 3H, CH_3_). ^13^C NMR (100 MHz, DMSO) *δ*_C_: 157.6, 148.8, 139.5, 135.4, 130.0, 129.2, 127.5, 124.7, 121.2, 119.6, 111.6, 56.6, 36.8. HRMS calcd for C_13_H_12_N_6_O_3_ ([M + Na]^+^): 323.0863. Found *m*/*z*: 323.0878.

#### *N*-(4-Methoxybenzyl)-3-methyl-4-oxo-3,4-dihydroimidazo[5,1-*d*][1,2,3,5]tetrazine-8-carboxamide (4i)

Off-white solid 199.7 mg (60% yield). Mp 193 °C. IR (cm^−1^) 3371 (N–H stretch), 3126 (C–H stretch), 1735 (C=O stretch, CONH), 1696 (C=O stretch, CONH). ^1^H NMR (400 MHz, DMSO-*d*_6_) *δ*_H_: 8.99 (t, *J* = 6.3 Hz, 1H, NH), 8.85 (s, 1H, CH), 7.27 (d, *J* = 8.8 Hz, 2H, ArH), 6.88 (d, *J* = 8.8 Hz, 2H, ArH), 4.42 (d, *J* = 6.3 Hz, 2H, CH_2_), 3.86 (s, 3H, CH_3_), 3.72 (s, 3H, CH_3_). ^13^C NMR (100 MHz, DMSO) *δ*_C_: 159.5, 158.1, 139.1, 134.5, 131.5, 130.3, 128.7, 128.4, 113.6, 55.0, 41.5, 36.1. HRMS calcd for C_14_H_14_N_6_O_3_ ([M + Na]^+^): 337.1020; found *m*/*z*: 337.1038.

#### 3-Methyl-*N*-(4-methylbenzyl)-4-oxo-3,4-dihydroimidazo[5,1-*d*][1,2,3,5]tetrazine-8-carboxamide (4j)

Off-white solid 168.5 mg (54% yield); mp 152 °C. IR (cm^−1^) 3397 (N–H stretch), 3102 (C–H stretch), 1736 (C=O stretch, CONH), 1665 (C=O stretch, CONH). ^1^H NMR (400 MHz, DMSO-*d*_6_) *δ*_H_: 9.01 (t, *J* = 6.4 Hz, 1H, NH), 8.86 (s, 1H, CH), 7.22 (d, *J* = 4.0 Hz, 2H, ArH), 7.12 (d, *J* = 4.0 Hz, 2H, ArH), 4.44 (d, *J* = 6.4 Hz, 2H, CH_2_), 3.86 (s, 3H, CH_3_), 2.27 (s, 3H, CH_3_). ^13^C NMR (100 MHz, DMSO) *δ*_C_: 159.6, 139.1, 136.5, 135.7, 134.5, 130.3, 128.7, 128.4, 127.3, 41.8, 36.1, 20.6. HRMS calcd for C_14_H_14_N_6_O_2_ ([M + Na]^+^): 321.1071; found *m*/*z*: 321.1090.

#### *N*-(4-Chlorobenzyl)-3-methyl-4-oxo-3,4-dihydroimidazo[5,1-*d*][1,2,3,5]tetrazine-8-carboxamide (4k)

Off-white solid 154.3 mg (47% yield). Mp 148 °C. IR (cm^−1^) – 3288 (N–H stretch), 1735 (C=O stretch, CONH), 1651 (C=O stretch, CONH). ^1^H NMR (400 MHz, DMSO-*d*_6_) *δ*_H_: 9.15 (t, *J* = 6.3 Hz, 1H, NH), 8.87 (s, 1H, CH), 7.41–7.33 (m, 4H, ArH), 4.47 (d, *J* = 6.3 Hz, 2H, CH_2_), 3.87 (s, 3H, CH_3_). ^13^C NMR (100 MHz, DMSO) *δ*_C_: 159.7, 139.1, 138.6, 134.6, 131.3, 130.1, 129.2, 128.5, 128.1, 41.4, 36.1. HRMS calcd for C_13_H_11_N_6_O_2_Cl ([M + Na]^+^): 341.0524; found *m*/*z*: 341.0542.

### Synthesis of analogues 3h–3n

TMZ acid (2, 200 mg, 1.03 mmol) was solubilised in mixture of THF and DCM (10 mL : 10 mL v/v). The desired alcohol was added (1.05 mmol) dropwise to the solution. The mixture was then stirred for 10 minutes. A solution of EDC·HCl (305 mg, 1.60 mmol) in DCM (2 mL) was added along with DMAP (1 mg). The reaction was stirred for 3 hours. The crude reaction mixture was embedded onto silica and subjected to flash column chromatography using a solvent system of an appropriate ratio of petroleum ether (40–60 °C) to ethyl acetate, to remove excess alcohol. The product was solubilised in DCM (20 mL) and washed with water (3 × 30 mL) and brine (30 mL). The DCM layer was dried over sodium sulphate and the solvent was evaporated under reduced pressure to afford the desired title compounds.

#### 4-Methoxyphenyl 3-methyl-4-oxo-3,4-dihydroimidazo [5,1-*d*][1,2,3,5] tetrazine-8-carboxylate (3h)

Off-white solid 489.8 mg (50% yield). Mp 172 °C; IR (cm^−1^) 3117 (C–H stretch), 1739 (C=O stretch)*. ^1^H NMR (300 MHz, DMSO-*d*_6_), *δ*_H_: 8.96, (s, 1H, CH), 7.22 (d, *J* = 9.0 Hz, 2H, ArH), 7.03 (d, *J* = 9.0 Hz, 2H, ArH), 3.92 (s, 3H, CH_3_), 3.79 (s, 3H, CH_3_). ^13^C NMR (75 MHz DMSO-*d*_6_), *δ*_C_: 159.0, 157.5, 143.8, 138.5, 136.3, 128.8, 128.6, 122.3, 114.5, 55.6, 36.8. HRMS calcd for C_13_H_11_N_5_O_4_ ([M + Na]^+^): 324.0703; found *m*/*z*: 324.0713. *Overlap of COO ester carbonyl and CO urea carbonyl.

#### 4-Nitrophenyl 3-methyl-4-oxo-3,4-dihydroimidazo [5,1-*d*][1,2,3,5] tetrazine-8-carboxylate (3i)

White solid 488.1 mg (49% yield). Mp 190 °C, IR (cm^−1^) 3154 (C–H stretch), 3114 (C–H stretch), 1754 (C=O stretch, COO), 1727 (C=O stretch, CONH). ^1^H NMR (300 MHz, DMSO-*d*_6_), *δ*_H_: 8.98 (s, 1H, CH), 8.38 (d, *J* = 9.0 Hz, 2H, ArH), 7.66 (d, *J* = 9.0 Hz, 2H, ArH), 3.94 (s, 3H, CH_3_). ^13^C NMR (75 MHz DMSO-*d*_6_), *δ*_C_: 158.6, 155.3, 145.8, 139.3, 138.3, 130.4, 126.0, 125.2, 123.7, 37.1. HRMS calcd for C_12_H_8_N_6_O_5_ ([M + Na]^+^): 339.0448; found *m*/*z*: 339.0460.

#### 4-Chlorophenyl 3-methyl-4-oxo-3,4-dihydroimidazo [5,1-*d*][1,2,3,5] tetrazine-8-carboxylate (3j)

White solid 442.3 mg (44% yield). Mp 164 °C; IR (cm^−1^) 3089 (C–H stretch), 1733 (C=O stretch)*. ^1^H NMR (400 MHz, DMSO-*d*_6_), *δ*_H_: 8,97 (s, 1H CH), 7.56 (d, *J* = 9.0 Hz, 2H, ArH), 7.38 (d, *J* = 9.0 Hz, 2H, ArH), 3.93 (s, 3H, CH_3_). ^13^C NMR (100 MHz DMSO-*d*_6_), *δ*_C_: 158.7, 148.8, 138.8, 137.5, 130.3, 129.8, 129.6, 125.2, 123.7, 36.6. HRMS calcd for C_12_H_8_N_5_O_3_Cl ([M + Na])^+^: 328.0208; found *m*/*z*: 328.0219. *Overlap of COO ester carbonyl and CO urea carbonyl.

#### 4-Cyanophenyl 3-methyl-4-oxo-3,4-dihydroimidazo[5,1-*d*][1,2,3,5]tetrazine-8-carboxylate (3k)

White solid 287.8 mg (30% yield). Mp 192 °C; IR (cm^−1^) 3146 (C–H stretch), 3110 (C–H stretch), 2224 (C≡N stretch), 1759 (C=O stretch, COO), 1730 (C=O stretch, CONH). ^1^H NMR (300 MHz, DMSO-*d*_6_), *δ*_H_: 9.00 (s, 1H, CH), 8.01 (d, *J* = 8.8 Hz, 2H, ArH), 7.58 (d, *J* = 8.8 Hz, 2H, ArH), 3.93 (s, 3H CH_3_). ^13^C NMR (75 MHz DMSO-*d*_6_), *δ*_C_: 158.2, 153.4, 138.8, 137.7, 134.2, 129.9, 124.8, 123.30, 118.3, 109.1, 36.6. HRMS calcd for C_13_H_8_N_6_O_3_ ([M + Na]^+^): 319.0550; found *m*/*z*: 319.0550.

#### Phenyl 3-methyl-4-oxo-3,4-dihydroimidazo[5,1-*d*][1,2,3,5]tetrazine-8-carboxylate (3l)

Off-white solid 75.3 mg (27% yield). Mp 149 °C; IR (cm^−1^) 3143 (C–H stretch), 1737 (C=O stretch)*. ^1^H NMR (400 MHz, chloroform-*d*), *δ*_H_: 8.53 (s, 1H, CH), 7.51–7.37 (m, 2H, ArH), 7.35–7.23 (m, 3H, ArH), 4.06 (s, 3H, CH_3_). ^13^C NMR (100 chloroform-*d*), *δ*_C_: 158.7, 150.2, 138.4, 136.3, 129.5, 128.8, 128.4, 126.3, 121.5, 36.8. HRMS calcd for C_12_H_9_N_5_O_3_ ([M + Na]^+^): 294.0597; found *m*/*z*: 294.0606. *Overlap of COO ester carbonyl and CO urea carbonyl.

#### 2-Methoxyphenyl 3-methyl-4-oxo-3,4-dihydroimidazo[5,1-*d*][1,2,3,5]tetrazine-8-carboxylate (3m)

Orange solid 150.7 mg (49% yield). Mp 165 °C; IR (cm^−1^) 3153 (C–H stretch), 1737 (C=O stretch)*. ^1^H NMR (400 MHz, DMSO-*d*_6_), *δ*_H_: 8.95 (s, 1H, CH), 7.31 (t, *J* = 7.6 Hz, 1H, ArH), 7.23 (ddd, *J* = 7.6, 8.2, 1.6 Hz, 2H, ArH), 7.04 (t, *J* = 7.6 Hz, 1H, ArH), 3.92 (s, 3H, CH_3_), 3.77 (s, 3H, CH_3_). ^13^C NMR (100 MHz DMSO-*d*_6_), *δ*_C_: 158.3, 150.8, 138.9, 137.4, 129.7, 127.3, 125.3, 122.8, 120.7, 112.9, 56.0, 36.5. HRMS calcd for C_13_H_11_N_5_O_4_ ([M + Na]^+^): 324.0703; found *m*/*z*: 324.0715. *Overlap of COO ester carbonyl and CO urea carbonyl.

#### *p*-Tolyl 3-methyl-4-oxo-3,4-dihydroimidazo[5,1-*d*][1,2,3,5]tetrazine-8-carboxylate (3n)

Off-white solid 81.5 mg (28% yield). Mp 164 °C; IR (cm^−1^) 3122 (C–H stretch), 1734 (C=O stretch)*. ^1^H NMR (400 MHz, chloroform-*d*) *δ*_H_: 8.51 (s, 1H, CH), 7.22 (d, *J* = 8.0 Hz, 2H, ArH), 7.17 (d, *J* = 8.0 Hz, 2H, ArH), 4.05 (s, 3H, CH_3_), 2.37 (s, 3H, CH_3_). ^13^C NMR (100 MHz, CDCl_3_) *δ*_C_: 158.8, 148.0, 138.4, 136.3, 136.0, 130.0, 128.8, 128.5, 121.2, 36.7, 21.0. HRMS calcd for C_13_H_11_N_5_O_3_ ([M + Na]^+^): 308.0754; found *m*/*z*: 308.0764. *Overlap of COO ester carbonyl and CO urea carbonyl.

### Cell lines, primary cells and culture conditions

Cytotoxic evaluation of the analogues was completed against 4 cell lines and 2 primary GBM cultures. The human glioma cell lines, GOS-3, U87-MG and 1321-N1 along with the human astroglial cell line, SVGp12, were purchased from The European Collection of Cell Cultures (ECCAC, UK) and Deutsche Sammlung von Mikroorganismen und Zellkulturen (DMSZ, Germany). Patient derived primary GBM cultures, BTNW374 and BTNW914, were obtained from The Brain Tumour North West Tissue Bank in collaboration with The Royal Preston Hospital. Patient data for primary cells was included as part of the ESI.†

1321-N1 (human astrocytoma) was maintained in DMEM medium supplemented with 2 mM l-glutamine and 10% FBS. GOS-3, (human brain mixed astro-oligodendroglioma) was maintained in DMEM medium supplemented with 2 mM l-glutamine and 10% fetal bovine serum albumin. U87-MG, (human glioblastoma) and SVGp12 (normal human astroglia) were maintained in EMEM medium supplemented with 2 mM l-glutamine, 1 mM sodium pyruvate and 10% FBS. All cell lines were incubated at 37 °C and 5% CO_2_ in a humidified incubator. Primary BTNW cultures were maintained in Ham's F10 nutrient mixture, containing 20 mM HEPES, 2 mM glutamine and 10% FBS. Primary cultures were incubated in a humidified incubator at 37 °C.

### CellTiter-Glo® assay

Cells were seeded in the appropriate media in each well of a Costar 96-well plate at a density of 5 × 10^2^ immortalised cells; 2 × 10^3^ (primary cells). The plates were incubated at 37 °C and 5% CO_2_ in a humidified incubator for 24 hours to allow cells to adhere. Each of the drugs tested were dissolved in DMSO to afford a 200 mM stock solution. The stock solutions were diluted with medium to afford the final working concentrations, ensuring DMSO concentration was no greater than 0.5% (v/v). Drug concentrations were added to the cells and incubated for 144 hours. After incubation, the drug concentrations were removed and replaced with 100 μL of CellTiter-Glo® and 100 μL of media. The luminescence of each well was measured on GENios Pro plate reader. The results for each of the drug concentrations were expressed as a percentage of the control wells. For cell lines, the regression equation for a polynomial line of best fit of order 2 was used to calculate the IC_50_. In the case of the *in vitro* model used in the present study, the IC_50_ was defined as the concentration of drug that results in a 50% reduction in oxyluciferin production. Primary culture cells were incubated with clinically relevant concentrations for 144 hours. Viability was expressed as a percentage of the control wells. Each experiment involving cell lines was carried out in triplicate of triplicates. Experiments using patient derived primary cultures were carried out in triplicate.

Initial screening of the 25 analogues was completed using the U87-MG cell line. The most promising analogues from the initial screening were taken forward and tested against the 1321-N1, GOS-3 and SVG-p12 cell lines. The results of all 4 cell lines were compiled and the analogues that were deemed to be the most promising drug candidates were tested against primary cultures at clinically relevant concentrations.

### General protocol for monitoring the stability of lead phenolic TMZ analogues on exposure to porcine liver esterase

A disodium phosphate, citric acid buffer (pH 6) was prepared by using disodium phosphate (Na_2_HPO_4_·12H_2_O, 200 mM) and citric acid (100 mM). A stock solution of 80 mg/100 mL of each of the TMZ esters (3h, 3i, 3j, 3k, 3l and 3n) were prepared in DMSO. The control reaction, without enzyme, was prepared by adding 3000 μL of buffer to the reaction vessel along with 56.25 μL of the stock solution. The test reaction, with enzyme, was prepared by adding 2999 μL of buffer to the reaction vessel along with 56.25 μL of TMZ ester. The final concentration of TMZ esters in the reaction vessels was 1.47 mg/100 mL. Porcine liver esterase (1 μL) was added to the test reaction at zero time (minutes). Both the control and test reactions were then incubated at 37 °C. Aliquots of 200 μL were transferred from the reaction vessels to 2 mL Eppendorf tubes, at 3 minute intervals for 30 minutes. The reaction was quenched by adding 200 μL of acetonitrile. The resulting samples were diluted with mobile phase and transferred to HPLC vials before analysis.

**Fig. 3:**
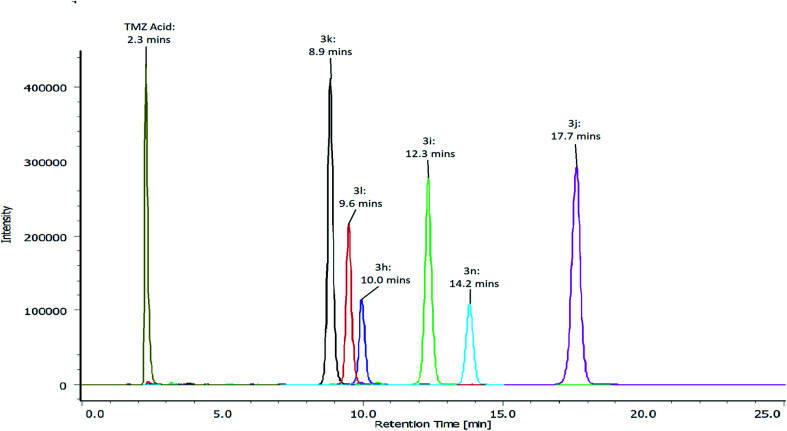
Chromatogram indicating the retention times of TMZ acid and ester analogues of TMZ 3h, 3i, 3j, 3k, 3l and 3n analysed using HPLC (sodium acetate buffer (20 mM) : acetonitrile 60 : 40, v/v, pH 4.5).

The chromatographic conditions: RP-HPLC, the mobile phase consisted of 60% sodium acetate buffer (20 mM) and 40% acetonitrile (pH 4.5). Analysis was performed at 1 mLmin^−1^ at ambient temperature on a Waters Symmetry Shield RP C_18_ column (4.6 mm × 250 mm), containing particles equivalent 5 micron. Detection of TMZ acid and TMZ esters (3h, 3i, 3j, 3k, 3l and 3n) was carried out at 325 nm. Detection of 4-methoxyphenol and 4-chlorophenol was carried out at 225 nm. Detection of 4-nitrophenol, 4-hydroxybenzonitrile, phenol and *p*-cresol was carried out at 325 nm, 250 nm, 270 nm and 225 nm, respectively (Fig. 3).

## Conclusion

The present work reports on an exciting series of phenyl ester TMZ analogues which possess enhanced cytotoxic potency against specified glioma and primary GBM cells. The *para*-substituted phenyl esters elicit significant cytotoxicity irrespective of MGMT expression in TMZ resistant patient derived primary cultures. The activity observed against MGMT expressing primary cultures led to the assumption that these analogues could possibly generate cytotoxicity *via* a mechanism of action distinct from methylation, however, this hypothesis needs to be further explored. Although this work is very much in its infancy, we believe that these analogues have the potential to act as superior chemotherapeutic agents for the treatment of GBM, eliciting a greater therapeutic response, compared to TMZ in a wider range of patients. The next stage in the investigation of the most promising analogues will centre around the use of an *in vivo* model to further assess their effectiveness. These investigations will initially focus on the bio-distribution and BBB evaluation before proceeding in assessing them against *in vivo* GBM.

## Conflicts of interest

There are no conflicts of interest to declare.

## Supplementary Material

RA-010-D0RA02686G-s001
