# Supplementary material for: A novel series of phenolic temozolomide (TMZ) esters with 4 to 5-fold increased potency, compared to TMZ, against glioma cells irrespective of MGMT expression
Source: RSC Adv. 2020 May 6;10(30):17561–70. doi: 10.1039/d0ra02686g (PMC9053583; doi:10.1039/d0ra02686g)
Supplement: RA-010-D0RA02686G-s001 [file RA-010-D0RA02686G-s001.pdf]

This journal is © The Royal Society of Chemistry 2020

Electronic Supplementary Information

ESI 1: Characteristics of patient-derived primary cultures used to assess the antiproliferative effects of TMZ analogues. ICH staining to assess MGMT status was carried out by pathology staff from The Royal Preston Hospital using the Allred scoring system.

**Patient-derived primary culture**	**BTNW914**	**BTNW374**
**Patient Gender**	Female	Female
**Patient Age**	65	33
**Tissue**	Brain	Brain
**Disease State**	Grade IV human glioblastoma	Grade IV human glioblastoma
**Survival Post Resection**	10 months	8 months
**All Red Score for MGMT (Status)[a]**	(PS: 0) + (IS: 0) = TS: 0 (Negative)	(PS: 5) + (IS: 3) = TS: 8 (Positive)

Table Footnotes

[a]Allred scores are expressed using both the proportional score (PS), (the proportion of MGMT expressing cells); and the intensity score (IS), (the average intensity of staining). The PS and the IS are then added together to give a total score (TS), the threshold for a sample to be deemed positive is >2.
